# Probable Posttraumatic Stress Disorder and Psychiatric Co-morbidity among Latino Primary Care Patients in Puerto Rico

**DOI:** 10.4172/2167-1044.1000124

**Published:** 2012-12

**Authors:** Mildred Vera, Deborah Juarbe, Norberto Hernández, Adriana Obén, Coralee Pérez-Pedrogo, William F Chaplin

**Affiliations:** 1 Center for Evaluation and Sociomedical Research, School of Public Health, Medical Sciences Campus, University of Puerto Rico, San Juan, Puerto Rico; 2 Medical Sciences Campus, University of Puerto Rico, San Juan, Puerto Rico; 3 Department of Psychology, St. John’s University, New York, USA

**Keywords:** Posttraumatic stress disorder, Latinos, Puerto Ricans, Primary care, Depression, Generalized anxiety disorder, Psychiatric co-morbidity

## Abstract

**Background:**

The present investigation was designed to study PTSD among inner city primary care patients in Puerto Rico. Specifically, we examined the rate of probable PTSD, PTSD co-morbidity with MDD and GAD, and the association of probable PTSD and co-occurring disorders with demographic, treatment, and alcohol related factors.

**Methods:**

We screened 3,568 patients at primary care practices serving primarily low-income patients. The presence of probable PTSD was assessed with the Primary Care PTSD screen, major depression with the PHQ-9, and generalized anxiety disorder with the GAD Q-IV.

**Results:**

Fourteen percent of our sample screened positive for probable PTSD. Among this group, 12% met criteria for co-morbid GAD without MDD and 15.9% for co-morbid MDD with/without GAD, whereas 72% of the patients with probable PTSD did not meet criteria for co-morbidity. Over 80% of the patients with probable PTSD indicated they were not receiving mental health treatment. Multiple logistic regression findings show that there were no significant differences in demographic and alcohol related factors by PTSD status. Multinomial logistic regression analysis revealed significant differences in the use of mental health treatment among the subgroups of patients with probable PTSD. As compared to patients with only probable PTSD, the use of mental health services was 4 times higher among patients with probable PTSD and MDD and over 2 1/2 times higher among patients with probable PTSD and GAD.

**Conclusion:**

The prevalence rate of probable PTSD in our sample was similar to the rates reported for soldiers after returning from deployment and for Latinos after the September 11 attacks. The high prevalence of probable PTSD and low use of mental health treatment among inner city primary care patients in our study, highlight the need of future research to obtain information on how to effectively target and treat Latino primary care patients in need of treatment for PTSD.

## Introduction

Posttraumatic Stress Disorder (PTSD) is a disabling anxiety disorder that may develop after exposure to an overwhelming traumatic event. Although PTSD is usually associated with military combat, it is common after sexual abuse, violent attacks (i.e. assault, rape, domestic violence), and catastrophes (i.e. harmful and fatal accidents, natural disasters, terrorism). PTSD affects approximately 7–9% of adult’s lifetime and about 4% currently [1–4] in the United States (US), while substantially lower prevalence rates have been reported in other counties [5–7]. When exposed to specific traumatic events some subpopulations appear to be at greater risk of PTSD. For example, findings from studies conducted after terrorist attacks [8–10], physical injury [11], police work [12,13], natural disasters [14], and military combat [15] suggest that Latinos in the US are at more risk for PTSD than their white and African American counterparts.

Latinos in the US comprise a heterogeneous group of persons from different Spanish-speaking cultures. When examined by subgroups, Puerto Ricans seem to be more vulnerable to PTSD than other Latino groups. Ortega and Rosenheck [16] reexamined data on Vietnam War veterans to explore PTSD among specific Latino subgroups. They found that Puerto Rican veterans had a higher probability of having PTSD than other Latino veterans. More recently, a representative survey conducted in New York City in the aftermath of the September 11 attacks showed that the prevalence of probable PTSD was higher among Dominicans (14.3%) and Puerto Ricans (13.2%) than among other Latinos (6.1%) [9].

PTSD is a particularly devastating disorder with considerable personal and societal costs. People suffering from PTSD tend to re-experience traumatic situations through disturbing nightmares, flashbacks, or persistent thinking about the event. If left untreated, PTSD can last for years and lead to a range of problems, including substance abuse, job loss, and suicide [17–19]. Failure to provide effective treatment places a toll on the individual in terms of decreased quality of life, as well as substantial costs to society associated with diminished productive capacity and welfare dependency [20]. Furthermore, people with PTSD are more likely to have high co-morbidity with other mental health conditions, poor physical health, high levels of somatic symptoms, and increased use of primary health care services [3,21–24].

Research efforts to detect mental health conditions in primary care have concentrated mainly on the study of depression [25–27]. Recently, PTSD and its co-morbidities has become a major focus of attention among primary care patients. Existing studies suggest that current PTSD is more prevalent among primary care patients, resulting in current PTSD rates ranging from 9% to 17%, and as high as 32% for particular samples [21–23,28–31]. Findings also show that co-morbid depression and Generalized Anxiety Disorder (GAD) are highly prevalent among patients with PTSD [32,33]. Although primary care is a key setting for detecting and treating PTSD, few patients are recognized [30,34].

The number of Latinos included in PTSD clinical and epidemiological studies in primary care is limited. Given previous research findings that suggest high PTSD rates among Latinos in community samples [8,9,12–14], particularly for Puerto Ricans [9], it is relevant that further research be undertaken to develop a greater understanding of PTSD among Latino primary care patients. As a first step in this direction, we studied probable PTSD among inner city Latino primary care patients in Puerto Rico. Specifically, we examined the rate of probable PTSD, co-morbidity with MDD and GAD, and the association of probable PTSD and co-occurring disorders with demographic, mental health treatment, and alcohol related factors.

## Methods

### Participants

Participants were recruited among patients presenting for medical services at 10 primary care clinics serving primarily low income patients in urban areas of San Juan, Puerto Rico. Based on their availability patients were approached by a research assistant while waiting for their medical appointment. Ninety three percent of the patients agreed to participate. They were eligible for the study if they were aged 18–65 years and spoke Spanish. Written informed consent was obtained after the purpose and procedures of the study were explained. Arrangements were made with nursing personnel to guarantee that participants did not miss their clinic appointment as a result of participating in the study. Interviews were conducted in private offices to ensure confidentiality. The study protocol was approved by the institutional review board.

### Data collection

Data were collected over an 18-month period in 2011–2012 by trained interviewers, using a computer-assisted structured interview in Spanish. The Primary Care PTSD (PC-PTSD) [35] screen items were used to assess the presence of probable PTSD. The PC-PTSD, developed by the National Center for PTSD for use in primary care settings, is one of the most widely used screens for PTSD [36]. Consistent with the DSM-IV-TR diagnosis, the PC-PTSD includes four questions that assess the presence of the four main PTSD symptom clusters (intrusive experiencing, avoidance behaviors, hyper vigilance, and emotional numbing). The questions read “In your life, have you ever had any experience that was so frightening or upsetting that in the past month you? a) Had nightmares about it or thought about it when you did not want to? b) Tried hard not to think about it or went out of your way to avoid situations that reminded you of it? c) Were constantly on guard, watchful, or easily startled? d) Felt numb or detached from others, activities, or your surroundings?” Participants receive one point for each item endorsed. Research findings show that cutoff scores of 2 or 3 positive responses on the PC-PTSD are suggestive of PTSD with sensitivities of 0 .73 and 0.78 and specificities of 0.88 and 0.87, respectively [35]. Although initially the Veterans Administration (VA) used a cut-off score of two or greater as indicative of a positive screening for probable PTSD, they later increased the threshold to three positive responses [37]. Similar results for the PC-PTSD have been confirmed among civilian primary care settings. A study that compared four PTSD screening tests in civilian primary care concluded that the PC-PTSD appeared to be the best screening test, showing correct classification for over 80% and good sensitivity (0.85) and specificity (0.82) with a cut-off score of three [29]. In the present study we used a cut-off score of three to estimate probable PTSD. Our result for the Cronbach’s alpha coefficient was 0.92.

The presence of major depression was assessed with the Patient Health Questionnaire depression module (PHQ-9) [38] designed for use in primary care settings. The PHQ-9 items are based on the nine diagnostic criteria for major depression disorder (MDD) in the DSM-IV. The PHQ-9 has been validated against the Diagnostic Interview Schedule in two large studies [38]. Each item asks patients to indicate the frequency with which they experienced the depressive symptom during the two weeks before the interview. Each item is scored on a scale from 0, not at all, to 3, nearly every day. A depression diagnosis requires that five or more items, including at least one of the first two questions (little pleasure, feeling depressed), are scored 3 (more than half the days) or 4 (nearly every day). Item 9 (suicidal thoughts) can also be endorsed with a score of 2 (several days). Psychometric studies [39,40] sustain the validity of the Spanish version of the PHQ-9. In our study the PHQ-9 had excellent internal consistency (Cronbach’s alpha=0.90).

The Generalized Anxiety Disorder Questionnaire-IV (GAD-Q-IV) [41], a nine item self-report measure, was used to assess the criteria for GAD as delineated in the DSM-IV. Most items are dichotomous and measure excessive and uncontrollable worry and related symptoms. The GAD-Q-IV was scored using the dimensional scoring system with a cut-off score of 5.7 to denote the presence or absence of GAD. Newman et al. [41] found that this scoring system had a high concordance with a diagnosis of GAD as determined by the Anxiety Disorders Interview Schedule for DSM-IV, kappa=0.67, with 88% of clinician-diagnosed participants correctly classified. The psychometric properties of the GAD-Q-IV for the Hispanic population have been supported with demonstrated sensitivity and specificity [42,43].

The 3-item Alcohol Use Disorders Identification Test consumption questions (AUDIT-C) [44] was used to assess hazardous drinking. The AUDIT-C was developed by the World Health Organization for the detection of hazardous alcohol use or at-risk drinking in primary care settings. Scores of 4 or more for men and 3 or more for women represent a positive screen. In previous research these cut-off scores resulted in a sensitivity of 0.86 among men and 0.60 among women and a specificity of 0.72 among men and 0.96 among women [44,45]. Good sensitivity (0.98) and specificity (0.91) were reported for the Spanish version of the AUDIT-C [46]. With only three items, the AUDIT-C has shown low internal consistency with an alpha of 0.56 [47]. In our study, Cronbach’s alpha coefficient was also 0.56.

Participants were asked whether they were currently receiving or had received treatment during the previous six months from a psychiatrist or psychologist. Demographic information including age, marital and employment status, gender, Latino subgroup, and education was also obtained.

### Analyses

We used descriptive statistics to describe the rates of probable PTSD and its co-morbidity with MDD and GAD in this sample. Bivariate analyses examined relationships among study variables using chi-square to test proportional differences on categorical measures. Cronbach’s alpha was calculated to assess internal consistency of the measures. Multivariate analyses involved two sets of group comparisons. In the first we used multiple logistic regressions to examine demographic, mental health treatment, and alcohol related factors among patients who differed in probable PTSD status. Next, among patients with probable PTSD we used multinomial logistic regression to simultaneously estimate the multivariate associations of three probable PTSD subgroups (probable PTSD only, probable PTSD and GAD only, and probable PTSD and MDD with or without GAD) with demographic, mental health treatment, and alcohol related factors. In this analysis, the group with probable PTSD only was used as the reference category to which the others were compared. We used SAS software, version 9.1 [48] to conduct all statistical analyses.

## Results

### PTSD and co-morbidity

Fourteen percent (n=509) of our sample of 3,568 patients in inner city primary care clinics screened positive on the PC-PTSD, suggesting probable PTSD. Among this group, 12% (n=61) met criteria for co-morbid GAD alone and almost 16% (n=81) for co-morbid MDD with or without GAD, whereas 72% (n=367) of the patients with probable PTSD did not meet criteria for co-morbidity with MDD or GAD.

### Demographic characteristics

Table 1 presents demographic characteristics for the entire sample and separately by PTSD status and probable PTSD co-morbidities. The mean age of patients participating in the assessment was 42.9 years. One third had not completed high school, 79% were female, 40% were married, and 40% were employed. Almost three fourths of the patients were Puerto Ricans, while the remaining were predominantly Dominicans. The rates of probable PTSD and co-morbidity with MDD and GAD were similar across demographic groups.

### Specialized mental health treatment

Table 2 shows that 80.9% of the patients with probable PTSD indicated that they had not received care from a psychologist or psychiatrist during the previous six months. In comparison to patients without probable PTSD (14%), the use of mental health treatment by patients with probable PTSD was significantly higher (19%). When we examined the use of specialized care among patients with probable PTSD grouped by co-morbidity, we found substantial differences among groups. The highest rate was reported by patients with probable PTSD and MDD, approximately 40% of this group reported having received care from a psychologist or psychiatrist during the previous six months. The group with probable PTSD and GAD was next, almost one fourth of the patients within this group received specialized mental health care. Fourteen percent of the patients with probable PTSD and no co-morbidity reported receiving mental health care.

### Alcohol use

About 9% of primary care patients assessed reported hazardous use of alcohol (Table 2). Similar rates were identified among patients with probable PTSD (8.6%) and those without probable PTSD (9.6%). When we examined alcohol use among patients with probable PTSD grouped by co-morbidity a marginally significant association (p=0.06) was evidenced. Hazardous drinking among patients with probable PTSD/GAD (16.4%) was almost double the report of patients with probable PTSD alone (7.9%) and almost triples the report of patients with co-morbid MDD (6.2%).

### Factors associated with probable PTSD

Table 3 presents results for the multiple logistic regression analysis conducted to examine the association of demographic, mental health treatment, and alcohol related factors with probable PTSD. When patients with probable PTSD and those without were compared, there were no significant differences between them with respect to demographic factors and alcohol misuse. However, significant differences were observed in their use of specialized mental health services. Patients with probable PTSD were approximately one and a half times (OR=1.40, 95% CI=1.09–1.80) as likely as patients negative to probable PTSD to have received care from a psychologist or psychiatrist during the past six months.

### Factors associated with probable PTSD co-morbidities

The results of the multinomial logistic regression analysis, classifying patients with probable PTSD into three mutually exclusive groups: 1) probable PTSD only (reference group), 2) probable PTSD and GAD only, and 3) probable PTSD and MDD with or without GAD are presented in table 4. Patients with co-morbid probable PTSD were similar to patients with probable PTSD only in demographic variables. After adjusting for demographic variables, our findings revealed significant differences in the use of specialized mental health treatment among groups. As compared to patients with only probable PTSD, the use of specialized mental health services was 4 times higher among patients with probable PTSD and MDD (OR=4.39, 95% CI=2.48–7.76) and over two and a half times higher among patients with probable PTSD and GAD (OR=2.78, 95% CI=1.38–5.62). Patients with probable PTSD and GAD were also approximately two and a half times as likely to be a hazardous drinker as patients with probable PTSD only. Meanwhile there were no significant differences in alcohol use between patients with probable PTSD alone and patients with co-morbid MDD.

## Discussion

The current research is one of the first to study PTSD among Latino primary care patients in Puerto Rico. In this large sample of primary care patients, assessments with the PC-PTSD yielded an estimate of probable PTSD prevalence of 14.3%. Our results show a high rate of current probable PTSD among patients in inner city primary care practices in Puerto Rico. These findings are consistent with a recent report from a study of 88,235 active and reserve soldiers that assessed probable PTSD using the same measure and cut-off score as our study. Milliken et al. [49] found that 14.3% of the reserve soldiers met criteria for probable PTSD several months after returning from deployment in Iraq or Afghanistan. Our data are also similar to findings from studies conducted after the September 11 attacks in New York showing a high prevalence of probable PTSD among Latino samples in both community (13.2% and 14.3% among Puerto Ricans and Dominicans respectively) [9] and primary care samples (15.0% for females and 9.5% for males) [10].

Findings from studies examining the relationship between PTSD status and demographic characteristics have shown varying results. In our study, no significant difference in the prevalence of PTSD was noted in terms of age, education, marital status, employment, or gender. Results from comprehensive population studies [3] have shown significant differences in rates of PTSD by gender, marital status, and age. In studies with primary care patients Gillock et al. [21] found differences in prevalence rates based on education and marital status, while Weissman et al. [10] identified a significant difference in rates among females. These investigators found that the high rate of PTSD among women was mediated by their socioeconomic conditions, such as living alone with limited education and income. In concert with this explanation, a possible interpretation of our findings is that inner city patients, who are at increased peril of exposure to traumatic events associated with poverty and interpersonal, political, and community violence may be at increased risk of PTSD, irrespective of their demographic characteristics. It has been noted that the chronic stress of poverty and inner city living resulting from poor quality housing, economic hardship in meeting daily needs, witnessing violent crimes, and high risk for direct victimization may overburden an individual’s psychological resources and increase the risk for PTSD [50].

Our study also provides key findings about co-morbidity and use of mental health services in our sample of Latino primary care patients with probable PTSD. Results show that 28% of the patients with probable PTSD endorsed at least one of the two conditions assessed, 16% complied with criteria for MDD with/without GAD and 12% met criteria for GAD. We also found that over 80% of study participants with probable PTSD had not received specialized mental health care in the previous six months. Our findings revealed that the presence of co-morbid psychiatric disorders was significantly associated with the use of mental health services. While only 14% of the patients with probable PTSD by itself reported receiving mental health care, this number increased to 25% among patients with probable PTSD/GAD and 40% among patients with probable PTSD/MDD. Considerable research data have shown that compared to PTSD by itself, co-occurring PTSD and MDD is associated with increased disability, worse physical health, more frequent use of primary care services, and greater symptom severity [51–56]. The increased burden experienced by patients with co-morbid MDD could help explain the higher use of specialized mental health services by this group. Another possible explanation is that the recent emphasis on the management of depression has contributed to increase the recognition and treatment of MDD [57,58]. On the other hand, it has been noted that when PTSD co-occurs with MDD the identification of PTSD as primary diagnosis might be complicated, reducing the possibilities of a correct diagnosis and proper treatment [59].

Our study has some limitations that should be considered when interpreting the results. First, patients were assessed for three mental health disorders. A more comprehensive evaluation would most likely result in higher rates of psychiatric co-morbidity. Second, representativeness is a shortcoming. The data presented were collected from a convenience sample of adults receiving care in primary care settings. Since there was no randomization there may be biases in the selection of potential respondents. Furthermore, participating clinics were located in inner city settings serving mainly socioeconomically disadvantaged patients. Lower socioeconomic status has been linked to a higher prevalence of mental illness [60]. Thus, our findings may not generalize to patients in other primary care settings. Third, the measures used to assess mental disorders in this study provided self-report data that are not equivalent to structured clinical interviews. It has been raised that data obtained through clinical interviews are more precise, while self report measures may misclassify individuals who do not have the condition and overestimate prevalence estimates [61]. Fourth, the problem of bias due to social desirability and stigma is a well known in this kind of studies. Data were collected by face-to-face interviews to help prevent non-participation. However, underreporting may result due to respondent’s unwillingness to report embarrassing behaviors. We cannot precisely determine the extent to which underreporting biased our results, however if this was the case the rates of probable PTSD would be higher than those reported.

In summary, our study findings evidence a high rate of probable PTSD and low use of mental health services in our sample of Latino primary care patients. We have shown that the prevalence rate of probable PTSD among inner city primary care patients in Puerto Rico is similar to the rates reported by soldiers after military assignment and Latinos after terrorist attacks. Among patients with probable PTSD, four out of every five were not receiving mental health treatment. Use of mental health services was particularly low for patients with probable PTSD by itself, whereas patients with a co-occurring psychiatric disorder were more likely to receive care. Overall, these results call for further research to obtain much needed information on how to effectively target and treat Latino primary care patients in need of treatment for PTSD.

## Figures and Tables

**Table 1:** Probable PTSD and comorbidities according to demographic characteristics.

	Total Sample (n=3568) %	Probable PTSD		Comorbidities (n=509)		
		Yes (n=509) %	No (n=3059) %	Probable PTSD Only (n=367) %	Probable PTSD and GAD only (n=61) %	Probable PTSD and MDD (n=81) %
Total		14.3	85.7	72.1	12.0	15.9
Gender Male Female	20.6 79.4	14.3 14.3	85.7 85.7	78.1 70.6	9.6 12.6	12.4 16.8
Age 18–35 years 36–50 years 51–65 years	33.1 32.7 34.2	13.4 14.3 15.1	86.6 85.7 84.9	72.2 71.8 72.3	10.7 14.4 10.9	17.1 13.8 16.8
Education ≤11 12 >12	35.5 33.7 30.8	14.5 14.2 14.0	85.5 85.8 86.0	75.0 71.4 69.5	9.8 14.0 12.3	15.2 14.6 18.2
Marital status Married Nonmarried	40.8 59.2	14.2 14.3	85.8 85.7	71.4 72.6	12.1 11.9	16.5 15.5
Employment status Yes No	40.0 60.0	14.6 13.4	85.4 86.6	71.7 73.4	10.9 15.3	17.4 11.3
Puerto Rican Yes No	74.1 25.9	14.6 13.4	85.4 86.6	71.7 73.4	10.9 15.3	17.4 11.3

**Table 2:** Probable PTSD and comorbidities according to mental health treatment and hazardous alcohol use.

	Total sample	Probable PTSD		Comorbidities (n=509)		
	(n=3568) %	Yes (n=509) %	No (n=3059) %	Probable PTSD Only (n=367) %	Probable PTSD And GAD only (n=61) %	Probable PTSD and MDD (n=81) %
Tota	3568	14.3	85.7	72.1	12.0	15.9
Mental health treatment Yes No	14.9 85.1	19.1 80.9	14.2 85.8*	13.6 86.4	24.6 75.4	39.5 60.5***
Hazardous drinker Yes No	9.4 90.6	8.6 91.4	9.6 90.4	7.9 92.1	16.4 83.6	6.2 93.8

**Table 3:** Multiple logistic regression analysis of factors associated with probable PTSD

Variables	Odds ratio (95% CI)	*P* value
Female	1.01 (0.80–1.28)	.909
Age	1.00 (1.00–1.02)	.063
Education	0.99 (0.97–1.03)	.915
Married	1.01 (0.84–1.23)	.899
Employed	1.03 (0.84–1.28)	.758
Puerto Rican	1.12 (0.89–1.41)	.344
Mental health treatment	1.40 (1.09–1.80)	.008
Hazardous drinker	0.94 (0.67–1.32)	.718

**Table 4:** Multinomial logistic regression comparing patient groups with co-morbid probable PTSD and probable PTSD only.

Variables	Probable PTSD/GAD OR (95% CI)	Probable PTSD/MDD OR (95% CI)
Female	1.85 (.86–3.92)	1.84 (0.93–3.62)
Age	1.00 (0.98–1.03)	1.00 (0.97–1.02)
Education	1.05 (0.96–1.14)	1.00 (0.93–1.09)
Married	1.04 (0.59–1.83)	1.21 (0.73–2.02)
Employed	1.49 (0.81–2.75)	1.02 (0.57–1.82)
Puerto Rican	0.63 (0.33–1.20)	1.26 (0.64–2.46)
Mental health treatment	2.78 (1.38–5.62)	4.39 (2.48–7.76)
Hazardous drinker	2.51 (1.11–5.66)	0.86 (0.31–2.38)

## Abbreviations

PTSD: Posttraumatic Stress Disorder
GAD: Generalized Anxiety Disorder
PC-PTSD: Primary Care PTSD screen
DSM-IV: Diagnostic and Statistical Manual of Mental Disorders, Fourth Edition
PHQ-9: Patient Health Questionnaire depression module
MDD: Major Depression Disorder
GAD-Q-IV: Generalized Anxiety Disorder Questionnaire-IV
AUDIT-C: Alcohol Use Disorders Identification Test consumption questions
